# A Novel *in vitro* Model Delineating Hair Cell Regeneration and Neural Reinnervation in Adult Mouse Cochlea

**DOI:** 10.3389/fnmol.2021.757831

**Published:** 2022-01-10

**Authors:** Wenyan Li, Yizhou Quan, Mingqian Huang, Wei Wei, Yilai Shu, Huawei Li, Zheng-Yi Chen

**Affiliations:** ^1^Department of Otolaryngology-Head and Neck Surgery, Graduate Program in Speech and Hearing Bioscience and Technology and Program in Neuroscience, Harvard Medical School, Boston, MA, United States; ^2^Eaton-Peabody Laboratory, Massachusetts Eye and Ear Infirmary, Boston, MA, United States; ^3^ENT Institute and Otorhinolaryngology Department of Eye & ENT Hospital, State Key Laboratory of Medical Neurobiology and MOE Frontiers Center for Brain Science, Fudan University, Shanghai, China; ^4^Institutes of Biomedical Sciences, Fudan University, Shanghai, China

**Keywords:** hair cell, adult, regeneration, novel model, cochlea

## Abstract

The study of an adult mammalian auditory system, such as regeneration, has been hampered by the lack of an *in vitro* system in which hypotheses can be tested efficiently. This is primarily due to the fact that the adult inner ear is encased in the toughest bone of the body, whereas its removal leads to the death of the sensory epithelium in culture. We hypothesized that we could take advantage of the integral cochlear structure to maintain the overall inner ear architecture and improve sensory epithelium survival in culture. We showed that by culturing adult mouse cochlea with the (surrounding) bone intact, the supporting cells (SCs) survived and almost all hair cells (HCs) degenerated. To evaluate the utility of the explant culture system, we demonstrated that the overexpression of *Atoh1*, an HC fate-determining factor, is sufficient to induce transdifferentiation of adult SCs to HC-like cells (HCLCs). Transdifferentiation-derived HCLCs resemble developmentally young HCs and are able to attract adult ganglion neurites. Furthermore, using a damage model, we showed that degenerated adult ganglions respond to regenerated HCLCs by directional neurite outgrowth that leads to HCLC-neuron contacts, strongly supporting the intrinsic properties of the HCLCs in establishing HCLC-neuron connections. The adult whole cochlear explant culture is suitable for diverse studies of the adult inner ear including regeneration, HC-neuron pathways, and inner ear drug screening.

## Introduction

Hearing deficits affect one in 500 newborn babies and half of the senior population over 70 years of age in the world (Morton and Nance, 2006; Muller and Barr-Gillespie, 2015). Loss of the auditory hair cells (HCs) that detect sound is the major cause of hearing loss. Furthermore, the mammalian cochlea completely lacks the capacity to regenerate HCs in adulthood, which leads to permanent deafness (Liu et al., 2012).

In adult lower vertebrates including zebrafish, amphibians, and birds, supporting cells (SCs) spontaneously proliferate and transdifferentiate into new HCs after the damage of existing HCs which leads to restoration of hearing (Corwin and Cotanche, 1988; Ryals and Rubel, 1988; Jones and Corwin, 1993; Dooling et al., 1997). To study regeneration in mammals, the neonatal mouse cochlear culture system has been developed and used extensively (Zheng and Gao, 1999, 2000; Zheng et al., 2000). As a result, signaling pathways involving mammalian cochlear HC regeneration by SC-to-HC transdifferentiation with potential in therapeutics have been identified in neonatal mice (Zheng and Gao, 2000, Shou et al., 2003; White et al., 2006; Diensthuber et al., 2009; Mizutari et al., 2013; Bramhall et al., 2014). A general approach to culture neonatal mouse cochleae involves the isolation of the sensory epithelium, which is cultured by adhering to the surface of a culture dish. A similar approach for adult cochleae requires the disruption of the ossified cochlear bone followed by removing the sensory epithelium for culture. Under this condition, the sensory epithelium inevitably dies shortly later. *In vivo* HC regeneration has been reported (Arnold et al., 2010; Walters et al., 2017; Kong et al., 2020). However, an *in vitro* culture system for adult mouse cochleae is yet to be established to allow studies ranging from regeneration and HC-neuron interactions to drug screening Chan and Rouse, 2016. The construction of transgenic animal models has become a dominant method to study those aspects in the adult mammalian inner ear, which is time-consuming in addition to other limitations. We hypothesized that the integral cochlear structure is important to the survival of the adult cochlear sensory epithelial cells. By culturing adult mouse cochlea with the intact bone, some sensory epithelial cells may survive long-term within well-preserved cochlear architecture suitable for the study of adult mammalian cochleae in culture.

In this study, for the first time, we developed a novel *in vitro* adult mouse cochlear culture system that includes the sensory epithelium with the surrounding bony structure. With the adult mouse cochlear explant culture system, a majority of adult SCs are viable in culture for up to 3 weeks. We further demonstrated the utility of the culture system in the regeneration of HC-like cells (HCLCs) through SC-to-HC transdifferentiation by overexpression of an HC induction gene *Atoh1*. We illustrated that the system can be used to study the auditory pathway by establishing connections between regenerated HCLCs and adult ganglion neurons in culture.

## Results

### Adult Mouse Cochlear Sensory Epithelium Degenerates Rapidly With the Conventional Culture Method

Few studies have been carried out successfully using cultured adult mouse cochleae due to the difficulty in retrieving the sensory epithelia from the ossified cochlea, especially those from the mid-to-base turn. In this study, we first tested the dissection of the adult cochlear sensory epithelium according to the protocol established for culturing neonatal mouse cochleae (Parker et al., 2010; Landegger et al., 2017). Only the apical region of the sensory epithelium could be preserved using this method (Supplementary Figures 1a1, a2). After 2 weeks in culture, the whole sensory epithelium folded and degenerated, as shown by the disruption of the overall structure, loss of all HCs, and severely reduced the number of SOX2^+^ SCs (Supplementary Figures 1b1–b3). Thus, using the conventional method, the adult cochlear sensory epithelium cannot be cultured long-term.

### Culture of the Intact Adult Cochlea

**FIGURE 1:**
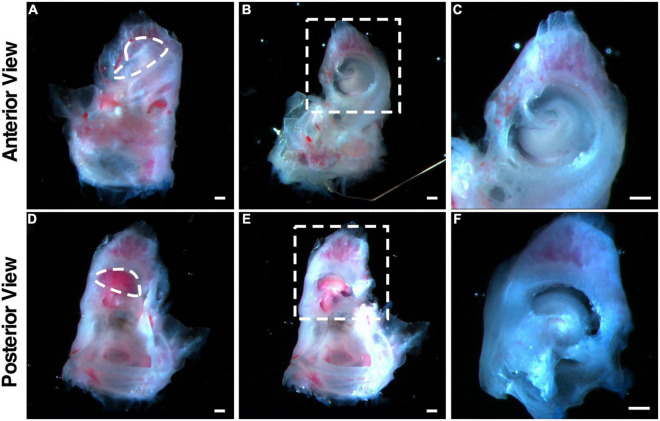
Illustration of the dissection procedure that maintains the architecture of the adult cochleae used in explant culture. **(A–C)** Freshly dissected adult mouse cochlea viewed from the anterior. The dotted circle in **(A)** indicates the openings to be created by removing the bony shell in the apex. **(B)** The cochlea after removing the bony shell with the exposure of the apex. **(C)** The enlarged image of the dotted box shown in **(B)**. **(D–F)** Freshly dissected adult mouse cochlea viewed from the posterior. The dotted circle in **(D)** indicates the openings to be created by removing the bony shell in the base. **(E)** The cochlea after removing the bony shell with the exposure of the base. **(F)** The enlarged image of the dotted box shown in **(E)**. Scale bars: 0.2 mm.

We reasoned that the degeneration and cell death in cultured adult cochlear sensory epithelia are due to the lack of the structure of the organ of Corti, which supports communications among cells and their environment and promotes cell survival. To test the hypothesis, we cultured adult (P30) mouse cochleae with the surrounding bone, which maintained the overall cochlear structure. We created openings, one in the apex and one in the base, and separated the entire cochlea from the vestibular part of the bony inner ear with forceps. Reissner’s membrane and the stria vascularis were carefully removed with a microprobe without touching the sensory epithelium, to expose the organ of Corti and allow the culture medium to pass through the endolymphatic space (Figure 1 and Supplementary Video 1).

**FIGURE 2:**
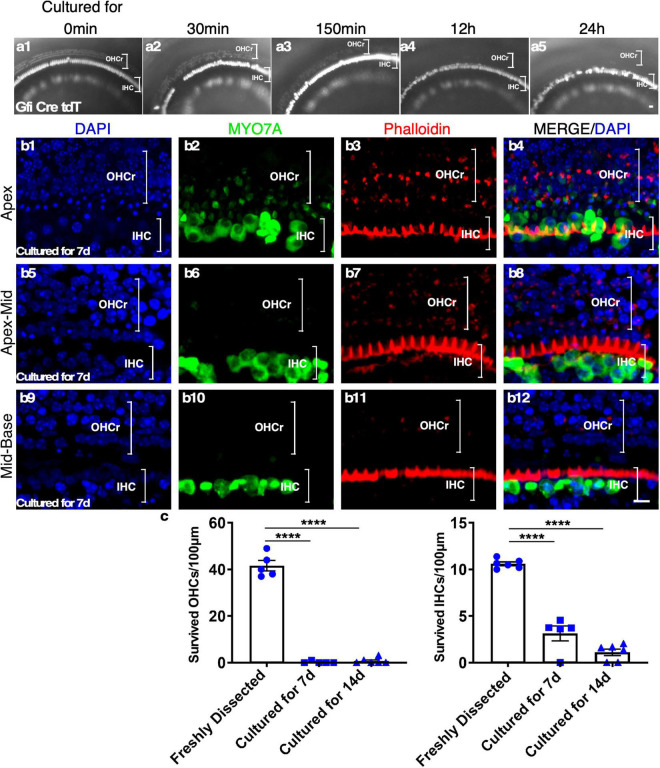
The rapid loss of outer hair cells (OHCs) in adult cochlear culture. **(a1–a5)** The loss of OHCs *in vitro*. In adult Gfi-Cre-Tm-red cochlear culture (apex), the loss of OHCs (by the absence of the Tm-red signals) started as early as 30 min in culture **(a2)**. By 12 h **(a4)**, no OHC signal was detectable, an indication of complete OHC loss. In contrast, many inner HCs (IHCs) survived 7 days in culture **(b1–b12)**. **(c)** Quantification of surviving OHCs and IHCs from apex to base of the adult cochlear culture system. ****p < 0.0001, two-tailed unpaired Student’s *t*-test. Error bar, mean ± SEM, *n* = 5–6. *n* is the number of biologically independent cochlea samples. Scale bars: 10 μm.

To characterize HC survival in cultured adult cochleae, we performed time-lapse recording using a reporter mouse model, *Gfi1*-Cre-Tm-red, in which tdTomato (Tm-red) expression was induced by *Gfi1* promoter-driven Cre activity (*Gfi1*-Cre). In *Gfi1*-Cre-Tm-red cochleae, Tm-red is induced in the cochlear HCs at the age of E12, with the Tm-red signal persisting throughout adulthood. Due to the difficulty of exposing the delicate structures of cultured adult sensory epithelia within the spatial bony structure, we evaluated the survival of the sensory epithelial cells based on the fluorescence signal (Tm-red). We observed rapid outer HC (OHC) death starting at 30 min from the beginning of the culture. The loss of OHCs continued and, by 24 h, all OHCs were virtually lost throughout the cochlea. To confirm the result, we stained existing HCs with Myosin VIIa (MYO7A)/Phalloidin 1 week after culture and noticed that nearly all OHCs died by this time point. Condensed and shrunken nuclei indicated dead OHCs, though some undegraded MYO7A protein could still be detected in the OHC region of the apex. In contrast, many inner HCs (IHCs) survived 1 week after culture, an indication of IHC resistance to cell death in culture (Figure 2).

**FIGURE 3:**
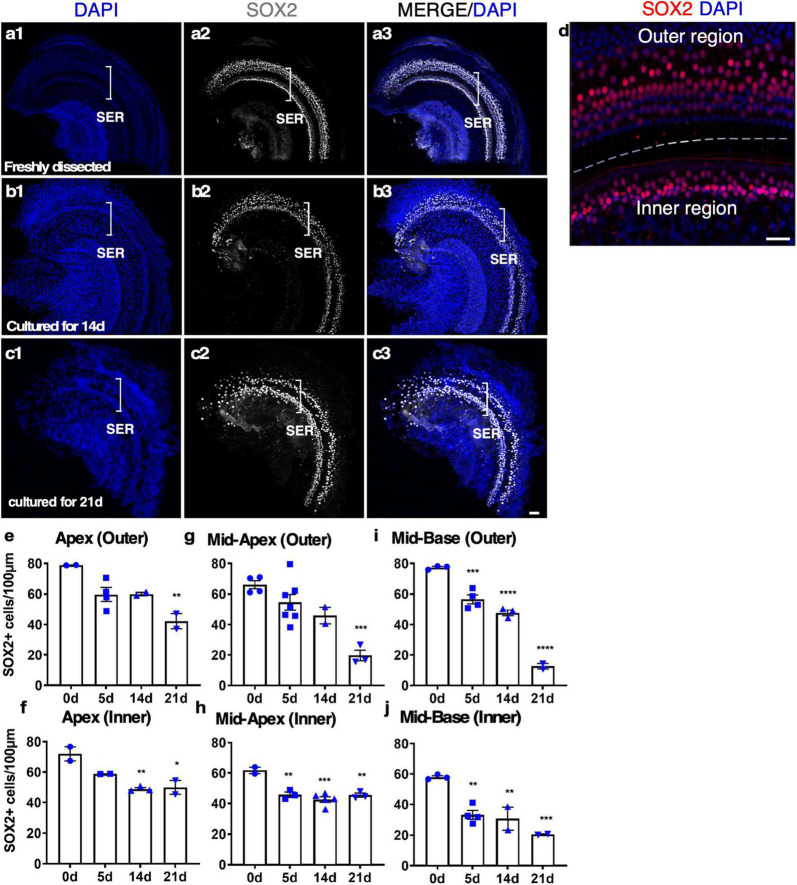
The survival of SOX2^+^ supporting cells (SCs) in adult cochlear explant culture. **(a–c)** Fresh and cultured wild-type adult cochlea (apex). **(a1–a3)** In a freshly dissected adult cochlea labeled with an anti-SOX2 antibody, SOX2^+^ SCs were confined to the sensory epithelial region (SER, bracket). **(b1–b3)** After 14 days in culture, the overall structure of the organ of Corti was intact with the survival of most of the SCs. **(c1–c3)** By 21 days, the loss of SOX2^+^ SCs became apparent yet the organ of Corti structure was maintained. **(d)** A freshly dissected sample to show the SCs in the inner and outer hair cell regions. **(e–j)** Quantification of SCs in the outer and inner SER (**d**, demarcated by a dashed line) along the cochlear turns showed similar patterns of survival of SCs in the apex and mid-turns for up to 14 days, and a diminished number of SCs in the base at 21 days in culture. *p < 0.05, **p < 0.01, ***p < 0.001, ****p < 0.0001. One-way ANOVA comparison of the data obtained on days 5, 14, and 21 with the data obtained day 0. Error bar, mean ± SEM, *n* = 2–7 in each group. *n* is the number of biologically independent cochlea samples. Scale bars: 50 μm.

To study SC survival in adult cochlear culture, we used a reporter mouse model, Sox2-CreER-Tm-red, in which *Sox2* promoter-driven Cre activity induces Tm-red after tamoxifen exposure (Sox2-CreER) in culture. After 14 days in culture, abundant SOX2^+^ cells were identified along the whole length of the cultured cochlear explant. Strong SC loss in the base and moderate SC loss in other regions only occurred at 21 days, indicating long-term survival of adult SCs in our culture system (Figure 3).

We studied the details of the cultured adult mouse cochleae from 5 to 21 days and found our whole cochlear explant culture method drastically improved the preservation of the overall structure of the organ of Corti and the integrity of the sensory epithelium. After 14 days in culture, the inner pillar cells (IPCs) and outer pillar cells (OPCs) were separated by the tunnel of Corti, similar to freshly dissected adult cochleae (Figures 3a, b and Supplementary Video 2). Due to HC death, SOX2 + SC nuclei could be seen as disorganized compared to a freshly dissected adult cochlea (Supplementary Video 2). We quantified the number of SCs in cultured adult cochleae. After 14 days in culture, ∼70% of SCs in the apex and ∼60% of SCs in the base survived, whereas, by 21 days, ∼50% of SCs in the apex and ∼13% in the base survived (Figures 3e–j), an indication of major SC loss in particular in the base between 14 and 21 days in culture. Approximately 30% of IHCs in the cultured adult cochleae could survive for 7 days, while only ∼10% of IHCs could survive for 14 days, and there is no significant difference between the cochlear turns. Nevertheless, even in the group with the longest culture time, the overall structure of the organ of Corti was maintained, in contrast to previous studies with a total loss of cochlear structure and SOX2^+^ SCs using the conventional method (Supplementary Figures 1b1–b3). We concluded that our new adult cochlear explant culture system maintains the overall structure of the organ of Corti with improved cell survival of SCs.

One important application of adult cochlear explant culture is the ability to study SCs in their relatively native status. To determine if adult SCs drastically change their properties, we examined multiple SC markers in the cultured adult cochlea. By immunolabeling with the SC markers of SOX2, JAG1, and S100A1, we found that the SCs in culture continued to express *Sox2.* JAG1 and S100A1 were detected in a subset of SCs in the cultured cochlea that resembled the patterns in the freshly dissected adult cochlea (Supplementary Figures 2a1–a3, c1–c3). There was a general diffusion of signals of JAG1 and S100A1 in cultured SCs, likely reflecting the disorganization of SCs after HC death (Supplementary Figures 2b1–b3, d1–d3).

### Expression of Exogenous Genes in Cultured Adult Cochlear Explants by a Viral Infection

**FIGURE 4:**
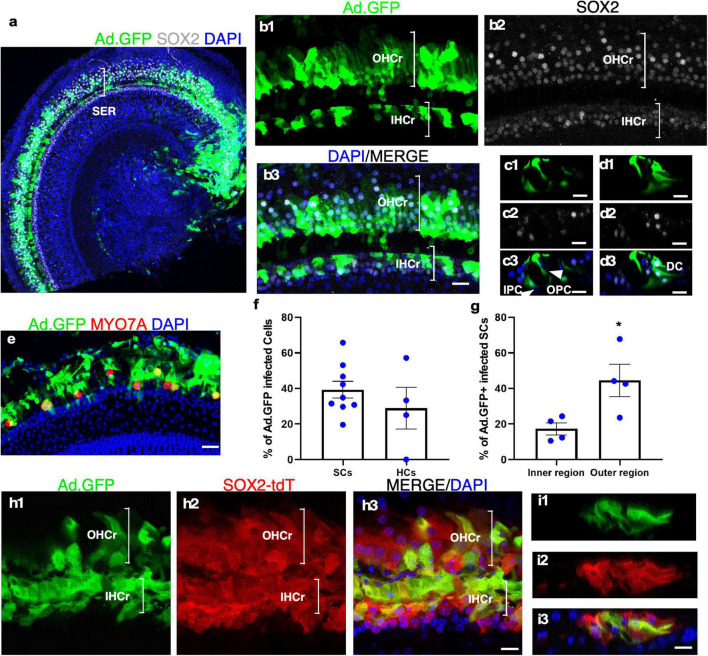
Adenovirus infects cultured adult SC subtypes and IHCs. **(a)** A surface view of a cultured adult cochlea (apical and part of the middle turn) infected by Ad-GFP with the production of GFP mainly in the SER with fewer infected cells in the limbus region. **(b1–b3)** The enlarged SER shows GFP^+^ SCs (SOX2^+^/GFP^+^). **(c,d)** Cross-sections of the SER to show the infected SC subtypes (SOX2^+^/GFP^+^) according to their positions in the organ of Corti, including inner and outer pillar Cells (IPCs and OPCs), Deiters’ cells (DCs), and inner phalangeal cells (IPhCs). **(e)** Surviving IHCs were infected by Ad-GFP (MYO7A^+^/GFP^+^). **(f,g)** Quantification and comparison of Ad-GFP-infected SCs vs. IHCs and infected SCs in the inner vs. outer SERs. **(h1–h3)** A lineage tracing study is shown in a cultured adult SOX2-CreER-Tm-red mouse cochlea treated by tamoxifen and infected by Ad-GFP. Tm-red and GFP were co-localized in the SCs in the sensory epithelium. **(i1–i3)** Cross-sections of the SER in **(h)** to show co-localization of Tm-red and GFP in the SCs. *p < 0.05, Student’s *t*-test. Error bar, mean ± SEM, *n* = 4–9. *n* is the number of biologically independent cochlea samples. Scale bars: 10 μm.

It has been reported that the recombinant, replication-deficient Adenovirus (Ad) could efficiently infect the HCs and SCs in the neonatal mouse cochlea (Shu et al., 2016). We investigated the ability of adenovirus to infect cultured adult cochleae to express exogenous genes. Two weeks after adding the adenovirus containing CMV promoter-driven GFP (Ad-GFP) to the culture media with adult mouse cochleae, we observed robust GFP signals in both the sensory epithelial and the spiral limbus regions (Figure 4a). In the sensory epithelial region, 39.36 ± 5.70% of the SOX2^+^ SCs were GFP^+^, whereas 28.87 ± 11.79% of the remaining MYO7A^+^ IHCs expressed GFP (Figures 4a–f). We further compared the infection efficiency among different SC subtypes by dividing SCs into the inner and outer regions that are separated by the tunnel of Corti (Figure 4d). The SCs in the inner region were composed of inner border cells (IBCs), inner phalangeal cells (IPhCs), and IPCs, while the SCs in the outer region consisted of OPCs, Deiters’ cells (DCs), Hensen cells and Claudius cells. Judging by the position, most SC subtypes including IPCs, OPCs, and DCs in cultured adult cochlear epithelia were infected by Ad-GFP (Figures 4c,d), and the infection efficiency of the SCs in the outer region was higher than that of the inner region (44.52 ± 9.06% in the outer region vs. 17.23 ± 3.39% in the inner region, *n* = 4, *p* < *0.05*) (Figure 4g). We further cultured adult *Sox2*-CreER-Tm-red cochleae in the presence of tamoxifen to activate Tm-red in SOX2^+^ SCs for lineage tracing. After Ad-GFP infection and tamoxifen treatment, Tm-red^+^ SCs were co-localized with GFP, demonstrating GFP expression in the SCs infected by Ad-GFP (Figures 4h,i). Efficient adenovirus-mediated SC infection and expression of exogenous genes in cultured adult cochlear explant provided an important tool to study gene functions.

### *In vitro* Regeneration of Hair Cell-Like Cells With *Atoh1* Overexpression in Cultured Adult Cochlear Explants

It has been established that overexpression of *Atoh1*, an HC fate determinant, is sufficient to transdifferentiate SCs to HCs in neonatal mouse cochleae and adult mouse vestibular system, yet transdifferentiation from SCs to HCs in adult cochleae *in vivo* remains controversial (Zheng and Gao, 2000; Kawamoto et al., 2003; Kelly et al., 2012; Liu et al., 2012; Yang et al., 2012). The capacity to culture adult mouse cochleae provides a unique opportunity to study adult HC regeneration *in vitro*. In adult cochleae cultured for more than 7 days, we did not observe any HCs in the OHC region due to the death of all OHCs in the culture and the lack of spontaneous HC regeneration. Rare IHCs survived in the culture (Figure 4e).

**FIGURE 5:**
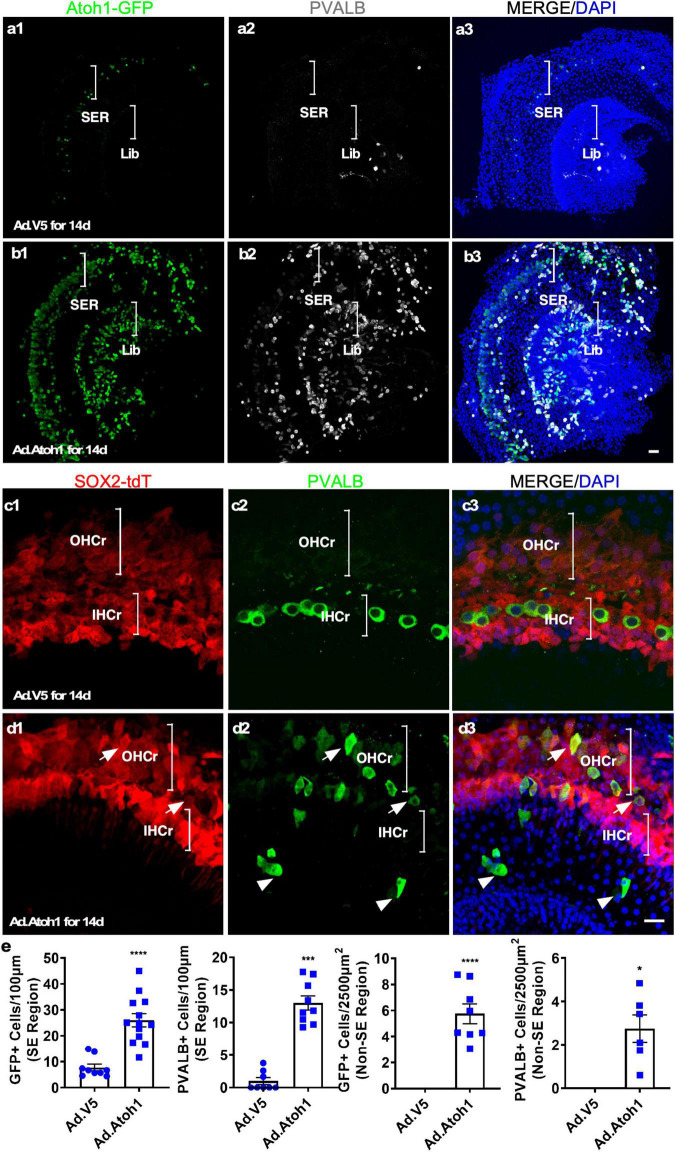
Transdifferentiation of Ad-Atoh1-infected cells and lineage tracing in cultured adult cochleae. **(a1–a3)** In cultured adult Atoh1-GFP cochleae (apex), control Ad-V5 infection-induced only a few GFP^+^ cells in the SER (bracket). There was no induction of GFP in the limbus region (Lib, bracket). **(b1–b3)** In cultured adult Atoh1-GFP cochleae (apex), Ad-Atoh1 infection-induced many GFP^+^ cells in the SER (bracket) and Lib (bracket). **(c,d)** SC origin of regenerated HC-like cells (HCLCs) by lineage tracing. **(c1–c3)** In the apex of the cultured adult Sox2-CreER-Tm-red cochleae infected with Ad-V5, no PVALB^+^ cells were detected in the OHC region whereas the surviving IHCs were Tm-red negative. **(d1–d3)** HCLCs (PVALB^+^) were detected in the OHC region that was co-localized with Tm-red signal (arrows) as well as in the non-sensory region (arrowheads) that were devoid of Tm-red after Ad-Atoh1 infection in cultured adult Sox2-CreER-Tm-red cochleae. **(e)** Quantification of GFP+ cells and new HCLCs from apex, apex-mid, and base in Ad-V5 and Ad-Atoh1 groups. *p < 0.05, ***p < 0.001, ****p < 0.0001, Student’s *t*-test. Error bar, mean ± SEM, *n* = 3–13. *n* is the number of biologically independent cochlea samples. Scale bars: 20 μm.

To test the utility of the explant culture system for HC regeneration *in vitro*, we cultured adult cochleae from the *Atoh1*-GFP transgenic mice, in which GFP expression was under the control of the 3′ enhancer of *Atoh1* (Lumpkin et al., 2003) and induced by *Atoh1* expression. We used adenovirus carrying CMV promoter-driven human *ATOH1* (Ad-Atoh1) to infect cultured adult *Atoh1*-GFP transgenic mouse cochleae, whereas adenovirus carrying a V5 tag (Ad-V5) was used as the control for infection. In Ad-V5-infected *Atoh1-*GFP cochleae that were subsequently cultured for 14 days, a few weak GFP^+^ cells were detected both in surviving IHCs and in the SCs vicinity of surviving IHCs in the explants (7.77 ± 1.30 GFP^+^ cells/100 μm, *n* = 9, Figures 5a1–a3 and Supplementary Figures 5a1–a4), reflecting the weak endogenous ATOH1 activity. In adult *Atoh1-*GFP cochleae infected with Ad-Atoh1 followed by culture for 14 days, quantitatively more GFP^+^ cells were detected throughout the sensory epithelium as well as in the spiral limbus region in the explants (26.07 ± 2.54 GFP^+^ cells/100 μm for sensory epithelium, *n* = 13, and 5.75 ± 0.76 GFP^+^ cells/2,500 μm^2^ for spiral limbus region, *n* = 11, Figures 5b,e). These GFP^+^ cells were the cells with *Atoh1* transcriptional activity due to Ad-Atoh1 infection.

To investigate whether cells with increased *Atoh1* activity transdifferentiate into HCs, we examined the co-expression of MYO7A and GFP in the infected adult cochlear explants. There were significantly more MYO7A^+^/GFP^+^ cells in the explants infected by Ad-Atoh1 (4.03 ± 0.66/100 μm sensory epithelium, *n* = 13, 0.64 ± 0.14 cells/2,500 μm^2^ spiral limbus region, *n* = 11, Supplementary Figures 3c1–c4) than in the explants infected by Ad-V5 (1.04 ± 0.47 cells/100 μm sensory epithelium, *n* = 9, Supplementary Figures 3a1–a4, b1–b4). We noticed that only a small portion of GFP^+^ cells turned out to be MYO7A^+^ HCLCs in both the sensory epithelial region and the limbus region after the infection of Ad-Atoh1 (16.00 ± 2.18% for the sensory epithelium, 12.57 ± 3.29% for the spiral limbus region) (Supplementary Figures 3d,e).

To further confirm the identity of regenerated HCs, we investigated the expression of the HC marker Parvalbumin (PVALB). After Ad-V5 infection of the cochlear explants from *Atoh1*-GFP transgenic mice, PVALB^+^ IHCs were observed in the sensory region of cultured cochlear explants, an indication of surviving existing IHCs (2.67 ± 0.61 cells/100 μm sensory epithelium, *n* = 3, Figure 5a), some PVALB^+^ spiral ganglion neurons (SGNs) were also found (Fischer et al., 2019). After Ad-Atoh1 infection, the number of PVALB^+^ cells was significantly increased in both the sensory epithelium (13.02 ± 1.076 cells/100 μm sensory epithelium, *n* = 9, Figures 5a, b and Supplementary Figures 5a, b) and the limbus region (2.76 ± 0.63 cells/2,500 μm^2^ limbus region, *n* = 6, Figures 5a, b). Of all the GFP^+^ cells, significantly more cells were PVALB^+^/GFP^+^ (60.64 ± 10.32% in the sensory region, *n* = 3 and 73.23 ± 11.81% in the limbus region, *n* = 3) than MYO7A^+^/GFP^+^.

To further determine the origin of HCLCs, we performed lineage tracing by using the *Sox2*-CreER-Tm-red mice. After injecting tamoxifen daily for 3 days, adult SCs were permanently labeled with tdTomato (Tm-red^+^). Cochleae from tamoxifen-injected adult mice were cultured and infected with either Ad-Atoh1 or Ad-V5. Fourteen days after Ad-V5 infection, we did not observe any PVALB^+^ HCLCs in the OHC region, and the remaining existing IHCs were negative for Tm-red (Figure 5c). In contrast, PVALB^+^ HCLCs were detected in the OHC region after Ad-Atoh1 infection and all of them were Tm-red^+^ (Figure 5d). We concluded that *Atoh1* overexpression resulted in regeneration of HCLCs in the OHC region from SOX2^+^ SC transdifferentiation and in the limbus region due to transdifferentiation of SOX2-negative cells.

### Regenerated Hair Cell-Like Cells Have Young Hair Cell Properties

**FIGURE 6:**
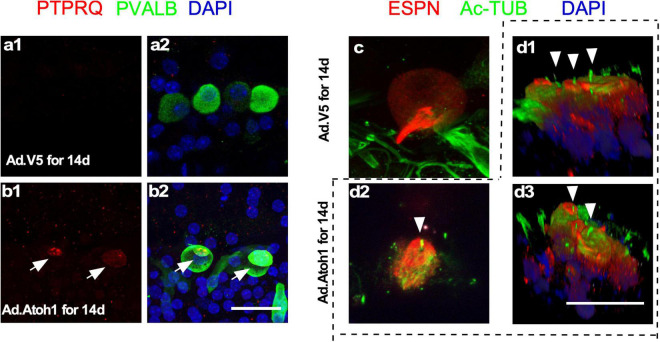
Regenerated HCLCs resemble developmentally young, immature HCs. **(a1,a2)** In control cultured adult cochleae infected with Ad-V5, an early HC marker PTPRQ was not detected in the existing surviving IHCs. **(b1,b2)** In Ad-Atoh1-infected adult cochleae in culture, PTPRQ was seen in the stereocilia structures in the regenerated HCLCs (arrows). **(c)** Kinocilia were not detected in control cochlear IHCs by acetylated tubulin (Ac-TUB) labeling after Ad-V5 infection in cultured adult cochleae. **(d1–d3)** Ac-TUB labeled kinocilia were observed in regenerated ESPN^+^ HCLCs after Ad-Atoh1 infection in cultured adult cochleae (arrowheads). Scale bars: 25 μm.

What are the characteristics of new HCLCs? Are they relatively mature HCs resulting from formation in the adult cochlea, or are they developmentally young HCs due to recent transdifferentiation from SCs? Using immunostaining with markers including PVALB, PTPRQ, espin (ESPN), and acetylated tubulin, we studied the presence of HC stereocilia in the regenerated HCLCs. Staining of PTPRQ, a marker for young HCs (Goodyear et al., 2003), was identified within the stereocilia-like structures of PVALB^+^ HCLCs, but not in the existing IHCs in control cochleae infected by Ad-V5 (Figures 6a,b). The kinocilium is a transient structure for directing the orientation of stereocilia during early cochlear HC development, and it is no longer detectable after postnatal day 21 (Cotanche and Corwin, 1991; Tilney et al., 1992). We observed acetylated tubulin-labeled kinocilia in the ESPN^+^ HCLCs in the Ad-Atoh1-infected cochlea but not in the existing IHCs from Ad-V5-infected control cochleae (Figures 6c,d). The detection of immature HC markers in the HCLCs but not in the existing surviving adult HCs strongly supports that regenerated HCLCs resemble developmentally young, immature HCs.

### The Survival of Adult Neurons in Cultured Cochlear Explants

**FIGURE 7:**
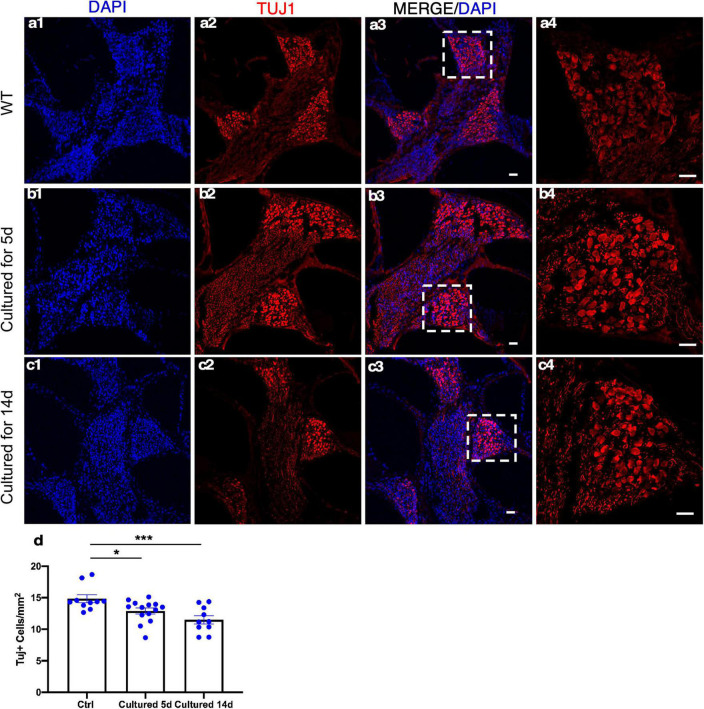
Spiral ganglion neurons (SGNs) survive in adult cochlear explant culture. **(a1–a4)** In the freshly dissected adult cochleae, SGNs in the modiolus were detected by TUJ1 labeling. After 5 **(b1–b4)** or 14 days **(c1–c4)** in culture, abundant TUJ1^+^ SGNs were seen in the modiolus with a well-maintained structure. **(d)** Quantification showed the survival of a majority of adult SGNs in the apical turn even after 14 days in culture. *p < 0.05, ***p < 0.001, and one-way ANOVA comparison of the mean of each column with the mean of a control column. Error bar, mean ± SEM, and *n* = 10–14. *n* is the number of biologically independent cochlea samples. Scale bars: 25 μm.

Spiral ganglion neurons relay the information generated by mechanotransduction of HCs to the brainstem. The ability to culture adult SGNs concomitantly with cochlear explants presents an opportunity to study SGNs and their interactions with the HCs that are essential in the auditory pathway for hearing. The whole cochlear explant culture enabled us to assess the survival of SGNs housed in the bony structure of the modiolus. We examined the density of SGNs within the modiolus by the labeling of TUJ1 (TUBB3), a neuronal marker, and quantified the number of TUJ1^+^ SGN somas from the frozen sections of cultured adult cochleae. Compared to the freshly dissected adult cochleae, 86 and 77% of TUJ1^+^ SGNs were maintained after 5 and 14 days in culture, respectively, demonstrating long-term survival of adult SGNs *in vitro* (Figure 7).

### Reinnervation in the Cultured Adult Cochleae

**FIGURE 8:**
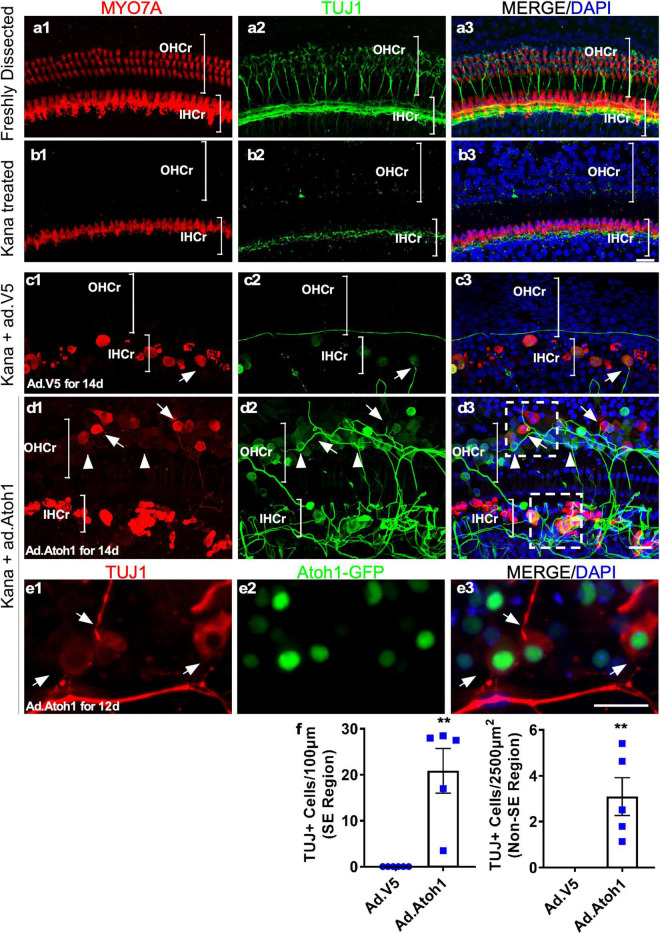
Promotion of outgrowth of adult SGN neurites and formation of connections with regenerated HCLCs in adult cochlear explant culture. **(a1–a3)** A freshly dissected apex of the adult cochlea showed the distribution of neurites labeled with TUJ1. **(b1–b3)** Nine days postkanamycin/furosemide injection *in vivo* (d0 in culture), there was a complete loss of OHCs and an absence of neurites in the OHC region (OHCr, bracket). Neurites with lower density were scattered beneath the surviving IHCs. **(c1–c3)** Nine days postkanamycin/furosemide injection *in vivo* and 14 days after Ad-V5 infection in culture (d14), adult cochleae showed no HCs or neurites in the OHC region. There was a loss of a majority of neurites in the IHC region (IHCr, bracket). Some surviving IHCs were in direct contact with remaining neurites (arrow). **(d1–d3)** Nine days postkanamycin/furosemide injection *in vivo* and 14 days after Ad-Atoh1 infection in culture, adult cochleae showed regenerated HCLCs and neurites in the OHC region, and numerous disorganized neurites in the IHC region. HCLCs (MYO7A^+^) were in direct contact with neurites (arrows). Many cells in the OHC region were TUJ1^+^ with weak or absent MYO7A signals, yet were in direct contact with neurites (arrowheads). The box areas in panel d3 refer to Supplementary Videos 3, 4. **(e1–e3)** Atoh1–GFP cochlea samples 12 days after Ad-Atoh1 infection. The Atoh1-GFP^+^ cells began to express TUJ1 marker and connect to neural fibers (arrows). **(f)** Quantification showed an increase of TUJ1^+^ cells after Ad-Atoh1 infection in culture. **p < 0.01, Student’s *t*-test. Error bar, mean ± SEM, *n* = 5–6. *n* is the number of biologically independent cochlea samples. Scale bars: 20 μm.

It has been established that after *in vivo* injection with ototoxic drugs including kanamycin in combination with diuretic drug furosemide, there is significant OHC loss that is accompanied by subsequent retraction of the ganglion neurites (Oesterle and Campbell, 2009). We used the model of kanamycin and furosemide injection in adult wild-type mice to induce OHC death. Examination of the cochleae 9 days post-injection showed a complete OHC loss and the absence of neurites in the OHC region (Figure 8b). In the IHC region, fewer neurites labeled with TUJ1 were observed medial to the IHCs in comparison to the freshly dissected untreated adult cochleae (Figures 8a,b).

We cultured adult wild-type cochleae 9 days postkanamycin/furosemide injection and infected one group with Ad-Atoh1 and the other group with Ad-V5 as control. In the Ad-V5-infected control after 14 days in culture, we observed surviving IHCs (MYO7A^+^) in the vicinity of the remnants of ganglion neurites, with occasional IHC to neurite connections (Figure 8c). No HCs or neurites were detected in the OHC region. In contrast, in the Ad-Atoh1-infected group, MYO7A^+^ HCLCs were detected in the OHC region (Figure 8d). Strikingly, numerous adult neurites migrated across the medial line to form connections with regenerated HCLCs in the OHC region. Furthermore, there were clusters of disorganized neurites surrounding the IHCs, many of which were in direct contact with the IHCs (Figure 8d and Supplementary Video 3).

We noticed that some regenerated MYO7A^+^ HCLCs in the OHC and IHC regions were also labeled with TUJ1, a neuronal marker. In fact, TUJ1^+^ cells were abundant in the sensory epithelia (20.87 ± 4.85/100 μm sensory epithelium, *n* = 5, Figures 8d,f) of the Ad-Atoh1-infected cultured adult cochleae. TUJ1 was mainly detected in cells with weak or absent MYO7A signal (arrowheads, Figure 8d), whereas cells with prominent MYO7A staining generally lacked TUJ1 (arrows, Figure 8d), suggesting that the expression of TUJ1 may be associated with early regenerated HCLCs transdifferentiated from SCs. Significantly, TUJ1^+^ cells were able to form connections with the SGN neurites irrespective of *Myo7a* expression (arrowheads, Figure 8d). To confirm the result, we treated the adult wild-type cochleae with Ad.Atoh1-mCherry, the adenovirus expressing both human *ATOH1* and mCherry marker under CMV promoter (Supplementary Figures 4b, 6). Notably, numerous neurites connected to Ad.Atoh1-mCherry^+^/TUJ1^+^ cells were detected in the culture system while no new TUJ1^+^ cells were detected in the Ad-V5 treated control samples (Supplementary Figures 4a1–a3, b1–b3). Besides, the triple labeling of Ad.Atoh1-mCherry^+^/ESPN^+^/TUJ1^+^ HCLCs was detected (Supplementary Figure 6). Furthermore, we infected Atoh1-GFP mouse cochleae with Ad-Atoh1 and detected GFP^+^ cells co-labeled with TUJ1. In addition, these GFP^+^/TUJ1^+^ cells form connections to the TUJ1^+^ SGN neurites (Figure 8e). The connections between neurites and regenerated HCLCs can be seen in a movie with the 3D-reconstructed images (Supplementary Videos 3, 4). Taken together, the results strongly indicated that the HCLCs promote neurite outgrowth and the likely formation of new connections between the SGN and HCs at an early stage of HC formation.

## Discussion

Embryonic or neonatal mouse cochleae can be cultured, which has led to studies to generate a wealth of information related to development, regeneration, and mechanisms underlying deafness (Zheng and Gao, 1999, 2000; Zheng et al., 2000; Shou et al., 2003; Woods et al., 2004; White et al., 2006; Dabdoub et al., 2008; Diensthuber et al., 2009; Mizutari et al., 2013; Bramhall et al., 2014). However, adult mature mammalian cochleae have characteristics that are fundamentally different from young cochleae. Since even newborn human inner ears are fully mature, studies in adult mature cochleae are necessary to understand disease progression, identify potential drugs, and develop regeneration therapy for the treatment of hearing loss. The lack of a system to study adult mammalian cochleae *in vitro* has been a major hurdle to efficiently test scientific hypotheses and screen new drugs for inner ear disorders. In this study, we established an adult mouse cochlear explant culture system and demonstrated its utility in a case study of HC regeneration and interaction between SGNs and HCs.

The major difference between our adult explant culture system and previous attempts to establish an adult cochlear explant culture lies in the fact that we purposefully retained the bony structure of the cochlea, maintaining cells in their endogenous environment that promotes cell survival. The overall architecture of the cochlea is important to cell survival as it is increasingly realized that mature cell types generally survive better in a 3D environment (Edmondson et al., 2014). Our culture system provides an integral architecture that sustains the relative positions of different adult cochlear cell types, which likely results in the maintenance of the cell-to-cell and cell-to-ECM interactions. The openings at the apex and the base of the cochlea facilitate access to the medium. Our results strongly support that a majority of adult cochlear cell types have the intrinsic capacity to survive long-term in explant culture, which allows the studies of individual cell types and their interactions.

In culture, adult OHCs undergo rapid cell death and, by 24 h, all OHCs are virtually lost (Figure 2). It is known that OHCs are sensitive to endolymph with high ionic concentration, and changes in the microenvironment lead to OHC death (Zenner, 1986; Dulon et al., 1987; Zenner et al., 1994). However, additional unknown factors are likely to contribute to cell death as OHCs die quickly even in artificial endolymph with a similar ionic environment to endogenous endolymph. Rapid OHC death in culture provides an opportunity to study HC regeneration in the OHC region as we have shown that all HCLCs in the OHC region are from regeneration rather than the survival of existing OHCs. The explant culture system should serve as an excellent model to screen potential drugs that attenuate OHC death in culture, which could lead to important hearing loss treatments by developing drugs that protect against HC death. In contrast to OHCs, some adult IHCs survive long-term (14 days) in culture, which may provide clues about mechanisms for their resistance to cell death. We are currently conducting experiments to profile gene expression in surviving IHCs by RNA-Seq to address the issue.

In the adult cultured explant, the overall cochlear structure is well maintained for up to 14 days (Supplementary Videos 1, 2) with the survival of a majority of SC types including Claudius cells, Hensen’s cells, DCs, pillar cells, IPhCs, IBCs, and inner sulcus cells. By studying SC markers, it is evident that SCs largely maintain their identities, i.e., SCs do not undergo a major dedifferentiation process with the perturbation of SC gene expression. Infection of diverse adult SC subtypes in culture by adenovirus provides an opportunity to activate or inhibit gene expression efficiently and temporally by methods, such as genome editing and siRNAs, and evaluate the effects on various activities including regeneration, treatment of genetic hearing loss, and SC biological roles (Shu et al., 2019; Niggemann et al., 2020).

In the current adult cochlear explant culture system, all OHCs and some IHCs, as well as a portion of SCs die. There is the reorganization of the sensory epithelium in cultured adult cochleae. Our explant culture system presents a damaged model. However, as it has been shown in our study, it can serve as a valuable tool to study different aspects of the adult cochlea in culture.

Reproducible regeneration of HCLCs in cultured adult cochlear explants by adenovirus-mediated *Atoh1* overexpression highlights the utility of the system. By activation of *Atoh1* in cultured adult mouse cochleae, we demonstrated that adult cochlear SCs can transdifferentiate into HCLCs, with the expression of multiple HC markers. In contrast, in our recent study, Ad-Atoh1 overexpression in adult cochlear SCs has never resulted in the regeneration of HCLCs *in vivo* (Shu et al., 2019). *In vivo* regeneration of HCLCs could only be achieved through reprogramming by transient activation of *Myc* and *Notch* followed by *Atoh1* overexpression (Shu et al., 2019). Therefore, it is highly likely that the explant culture itself provides a degree of reprogramming that enables adult SCs to respond to *Atoh1* induction signals and transdifferentiate to HCs *in vitro.* This is consistent with the evidence that mature cell types undergo reprogramming in culture by selective downregulation of mature genes and upregulation of developmental genes (Alizadeh et al., 2001; Mantikou et al., 2016). The cochlear explant culture system further illustrates that HCLCs can be regenerated from non-sensory epithelial regions, such as the limbus, providing evidence that additional adult cochlear non-sensory cell types have the capacity to transdifferentiate into HCs in culture in the presence of *Atoh1* signaling. Identification of signaling pathways in culture-mediated reprogramming in adult SCs should shed light on the process with potential improvement in HC regeneration efficiency.

Our study is the first in which adult SGNs were cultured for an extended period of time and were studied in the context of degeneration and reinnervation. In adult cochleae, the loss of HCs leads to retraction of neurites (Sugawara et al., 2005; Kujawa and Liberman, 2009). Furthermore, damage to HCs leads to the loss of synapses by the way of synaptopathy that contributes to “hidden hearing loss,” the reduced capacity to recognize speech in noisy environments. Efforts to reestablish new synapses after HC noise damage, including NT3 overexpression, have had limited success (Wan et al., 2014). However, it is not known if adult SGNs retain their potential for outgrowth and reestablishment of connections with HCs once they are retracted as the consequence of HC damage or death. The adult cochlear explant culture demonstrates the feasibility of adult SGN outgrowth which likely results in reestablishing connections with regenerated HCLCs. Future studies will involve optimization of the culture condition so that the culture time can be further extended, which would allow for the study of the behavior of type I and II ganglion neurons and synaptogenesis in adult cochleae *in vitro*.

Insightfully, we found that during HC regeneration, Ad-Atoh1-infected cells start to express *Tuj1*, a neuronal marker, before the expression of HC genes including *Myo7a.* At this stage, TUJ1^+^ neurites have migrated toward the TUJ1^+^ cells induced by Ad-Atoh1 infection to form likely connections (Figures 8d,e, Supplementary Figure 4, and Supplementary Videos 3, 4). Thus, the signals that promote neurite outgrowth and formation of connections may have been produced at a very early stage during HC regeneration. Identification of the signals responsible for the process will have a major impact on neurite outgrowth and synaptogenesis that are essential in developing potential therapy to reconnect SGNs with existing or regenerated HCs. A previous study on spontaneous HC regeneration in chicks has also identified early new HCs labeled with TUJ1 (Stone and Rubel, 2000), which is consistent with our observations during HC regeneration in adult cochleae *in vitro*. Expression of *Tuj1*, however, is transient, in regenerated HCLCs expressing mature markers, such as *Myo7a*. We noticed that there were only a small number of TUJ1+ cells that weakly express HC markers, such as Myo7a, and that these may represent prosensory cells before they more strongly express Myo7a and become HCLCs. For example, the triple labeling of Ad.Atoh1-mCherry+/ESPN+/TUJ1+ HCLCs was detected (Supplementary Figure 6). Furthermore, we infected Atoh1-GFP mouse cochleae with Ad-Atoh1 and detected GFP+ cells co-labeled with TUJ1. In addition, these GFP+/TUJ1+ cells form connections to the TUJ1+ SGN neurites (Figure 8e). The intermediate TUJ1 expression in early regenerated HCLCs may be unique to HC regeneration, as TUJ1 is not involved in normal HC development (Stone et al., 1996; Molea et al., 1999). Our data illustrate the application of the adult cochlear explant culture system to uncover novel biological processes.

Recent progress with inner ear organoids has provided new opportunities to study inner ear cell types including HCs *in vitro* (Koehler et al., 2013, 2017). The adult cochlear explant culture system is complementary to inner ear organoids for the study of the inner ear *in vitro.* The explant culture system uses adult cochleae, thus providing critical information on the capacity of mature inner ear cells to regenerate and develop cell-to-cell interactions. Furthermore, the regenerated HCs have properties that resemble the auditory HCs since the explant culture system uses adult cochleae. This has been illustrated by our recent study of Myc/Notch activation in combination with HC regeneration that leads to the production of HCLCs expressing *Slc26a5* (Prestin) and *Slc17a8* (Vglut3), an OHC marker and an IHC marker, respectively (Shu et al., 2019). The ability to study adult whole cochleae in their native architecture and in the presence of diverse inner ear cell types (cells from sensory and non-sensory regions as well as the SGNs) provides a unique opportunity to build an auditory system *in vitro.* It is possible that our approach can be extended to other mammalian species including pigs and humans so that the study can be carried out with results that are directly relevant to clinical applications.

## Materials and Methods

### Mouse Models

Sox2-CreER transgenic mice and tdTomato reporter mice were from Jackson Laboratory (Stock# 017593 and 007914, respectively); We also used a strain expressing tdTomato under the control of *Gfi1* promoter (Gfi1 ^TM 1(Cre)Gan^; R26tdTomato). *Atoh1*-nGFP mice are from Jane Johnson, University of Texas Southwestern Medical Center, Dallas, TX, United States. The wild-type mice were C57BL/6 from Charles River Laboratories. All experiments were performed in compliance with ethical regulations and approved by the Animal Care Committees of Massachusetts Eye and Ear and Harvard Medical School.

### Adult Cochlear Culture and Viral Infection *in vitro*

Different from the neonatal cochlea culture method, in which the cochleae were disassociated from the bone, adult mouse cochleae (6–8 weeks old) were dissected with the bone attached. The bulla was first removed from the skull and then, in brief, dipped in 70% ethanol before being placed in ice-cold Hanks’ Balanced Salt Solution (HBSS). The vestibular region was also removed. Under a dissecting microscope, the middle ear, vessels, and debris were removed from the bulla. The bone covering the apical turn was chipped off, and round window and oval window membranes were opened to allow media exchange with the cochlea fluids. The ligament portion and Reissner’s membrane at each end of the cochlea were also removed to facilitate the access of medium to the sensory cells. The cochleae were maintained in floating culture in DMEM/F12 (Invitrogen) supplemented with N2 and B27 (both from Invitrogen) for 5–21 days. For infection, adenovirus was added to the culture at a titer of 5 × 10^10^ pfu/ml overnight before replacement with fresh medium. Ad-*Atoh1* and Ad-*V5* were purchased from SignaGen Laboratories, Rockville, MD, United States. Ad-GFP was purchased from the Vector Lab, Baylor College of Medicine, Houston, TX, United States. To label proliferating cells, EdU was added at a final concentration of 10 μM for varying time points.

### Lineage Tracing

Notably, 6-week-old Sox2-CreER/tdT*^f/f^* mice were injected with tamoxifen (75 mg/kg) daily for 3 days before the cochleae were dissected for culture. Each virus was added to the medium for 16 h at a concentration of 5 × 10^10^ pfu/ml.

### Immunohistochemistry

Mouse cochleae were fixed in 4% paraformaldehyde at 4°C overnight, followed by decalcification in 120 mM EDTA for 1–2 days. The decalcified cochleae were used for whole-mount immunohistochemistry following a standard procedure (Huang et al., 2013). The antibodies used are shown in Supplementary Table-antibody.

### Confocal Microscopy

Confocal microscopy was performed using a Leica TCS SP8 with Leica Application Suite Advanced Fluorescence (LAS AF) software version 2.6.0 (Leica Microsystems Inc., Buffalo Grove, IL, United States). Sequential scanning with different laser channels was used for image acquisitions. Confocal images were processed using the ImageJ package (National Institutes of Health, Bethesda, MD, United States)^1^. For Z-stacks, equal numbers of images of adjacent optical sections, 0.5 μm in thickness, were used for processing with identical parameters, including median filtering and adjustment of brightness and contrast, between experimental and control groups.

### Statistical Analysis

The Prism 8 statistical package (GraphPad Software, Inc., San Diego, CA, United States) was used in the data processing. The number of SOX2^+^ cells was counted as an SC number. The average number of SOX2^+^ SCs per 100 μm was calculated as SC density for each cochlea and was used for statistical analysis. Data were presented as mean ± SEM. ANOVA analysis with Tukey’s multiple comparisons test was used to compare three or more groups (*p* < 0.05 was considered significant).

## Data Availability Statement

The original contributions presented in the study are included in the article/Supplementary Material, further inquiries can be directed to the corresponding authors.

## Ethics Statement

The animal study was reviewed and approved by the Massachusetts Eye & Ear Infirmary IACUC.

## Author Contributions

WL and YQ designed and performed the experiments, analyzed the data, interpreted the results, and drafted the manuscript. MH, WW, and YS performed the experiments and analyzed the data. HL analyzed the data. Z-YC conceived the project, designed the experiments, analyzed the data, interpreted the results, and wrote the manuscript. All authors edited the manuscript.

## Conflict of Interest

Z-YC has a financial interest in Salubritas Therapeutics, LLC, which is developing treatments for hearing loss including genome editing, inner ear regeneration, novel delivery, and gene therapy. The remaining authors declare that the research was conducted in the absence of any commercial or financial relationships that could be construed as a potential conflict of interest.

## Publisher’s Note

All claims expressed in this article are solely those of the authors and do not necessarily represent those of their affiliated organizations, or those of the publisher, the editors and the reviewers. Any product that may be evaluated in this article, or claim that may be made by its manufacturer, is not guaranteed or endorsed by the publisher.

## Footnotes

1
https://imagej.nih.gov/ij/

## Supplementary Material

Supplementary Figure 1 Adult cochlear sensory epithelium degenerates in culture with the conventional method. **(a1,a2)** Epifluorescent picture of the apical turn of the freshly dissected wild-type adult mouse cochlea according to the protocol established for culturing neonatal mouse cochlea (Parker et al., 2010; Landegger et al., 2017). Only the very apical region of the sensory epithelium could be preserved with this method. Panel **(a2)** is the box area in panel **(a1)**, with the sensory epithelial regions (SER) marked with a bracket. **(b1–b3)** Fluorescent picture of the apical turn of an adult mouse cochlea using the traditional culture method. After 2 weeks in culture, the whole sensory epithelium folded and degenerated, with the loss of virtually all SOX2^+^ cells. Scale bars: 50 μm.

Supplementary Figure 2 Maintenance of supporting cell (SC) markers in cultured adult cochleae. **(a1–a3)** In a freshly dissected adult cochlea (middle turn), markers SOX2 and JAG1 were co-localized in the SCs. **(b1–b3)** After 5 days in culture, a subset of SOX2^+^ cells was co-labeled with JAG1. Notice disorganization of SCs compared with the freshly dissected sample. **(c1–c3)** Co-localization of SOX2 and S100A1 in a subset of SCs of a freshly dissected adult cochlea. **(d1–d3)** SOX2 and S100A1 were similarly co-localized in a subset of SCs of adult cochleae after 5 days in culture. Again, the disorganization of SCs was evident in culture. Scale bars: 50 μm.

Supplementary Figure 3 *In vitro* regeneration of hair cell-like cells (HCLCs) with *Atoh1* overexpression. **(a1–a4)** In cultured adult *Atoh1*-GFP cochleae infected with Ad-V5, a few GFP^+^ cells were observed mainly in the existing inner HCs (IHCs), but not in other sensory epithelial regions. In cultured adult *Atoh1*-GFP cochleae infected with Ad-Atoh1, increased numbers of GFP^+^ cells were observed across the sensory epithelial region **(b1–b4)** and the limbus region **(c1–c4)**. Some GFP^+^ cells were co-labeled with MYO7A, indicating regeneration of HCLCs in those areas. **(d,e)** Quantification data showed more MYO7A^+^ HCLCs that were detected in both the sensory epithelial region and the limbus region in cultured adult *Atoh1*-GFP cochleae infected by Ad-Atoh1 than by Ad-V5 infection. ***p* < 0.01, ****p* < 0.001, Student’s *t*-test. Error bar, mean ± SEM, *n* = 9–13. *n* is the number of biologically independent cochlea samples. Scale bars: 20 μm.

Supplementary Figure 4 Atoh1 overexpression generates TUJ1^+^ cells in adult cochlear explant culture. **(a1–a3)** Nine days postkanamycin/furosemide injection *in vivo* and 12 days after Ad-V5 infection in culture (d12), and there was a loss of a majority of neurites in the IHC region (IHCr, bracket). **(b1–b3)** Nine days postkanamycin/furosemide injection *in vivo* and 12 days after Ad-Atoh1-mCherry infection in culture, adult cochleae showed regenerated TUJ1^+^/mCherry^+^ cells in the OHC region (OHCr, bracket), and significantly more neurites in the IHC region. TUJ1^+^ cells were co-labeled with mCherry (arrows). Scale bars: 20 μm.

Supplementary Figure 5 *In vitro* regeneration of HCLCs with *Atoh1* overexpression. Enlarged picture of the apex of a cultured adult *Atoh1*-GFP cochlea infected with Ad-V5/Ad-Atoh1. The samples were incubated with antibodies against GFP/MYO7A/PVALB. **(a1–a4)** In cultured adult *Atoh1*-GFP cochleae infected with Ad-V5 (the enlarged image of Supplementary Figure 3a), a few weak GFP^+^ cells were observed mainly inside and surrounding the existing IHCs, indicating the weak endogenous ATOH1 activity. **(b1–b4)** Many strong GFP^+^ cells were observed across the sensory epithelial region. Most GFP^+^ cells were co-labeled with PVALB. Scale bars: 10 μm.

Supplementary Figure 6 Atoh1 overexpression generates TUJ1^+^/ESPN^+^ HCLCs in adult cochlear explant culture. **(a1–a4)** (Low magnification) and **(b1–b4)** (high magnification). Twelve days after Ad-Atoh1-mCherry infection of cultured WT adult cochleae, regenerated mCherry^+^/TUJ1^+^/ESPN^+^ HCLCs in the sensory region of apex were detected. Atoh1mCherry^+^/TUJ1^+^ cells were co-labeled with an HC marker ESPN (arrows). Scale bars: 10 μm.
