# Supplementary figures and images for: A Novel in vitro Model Delineating Hair Cell Regeneration and Neural Reinnervation in Adult Mouse Cochlea
Source: Front Mol Neurosci. 2022 Jan 10;14:757831. doi: 10.3389/fnmol.2021.757831 (PMC8785685; doi:10.3389/fnmol.2021.757831)

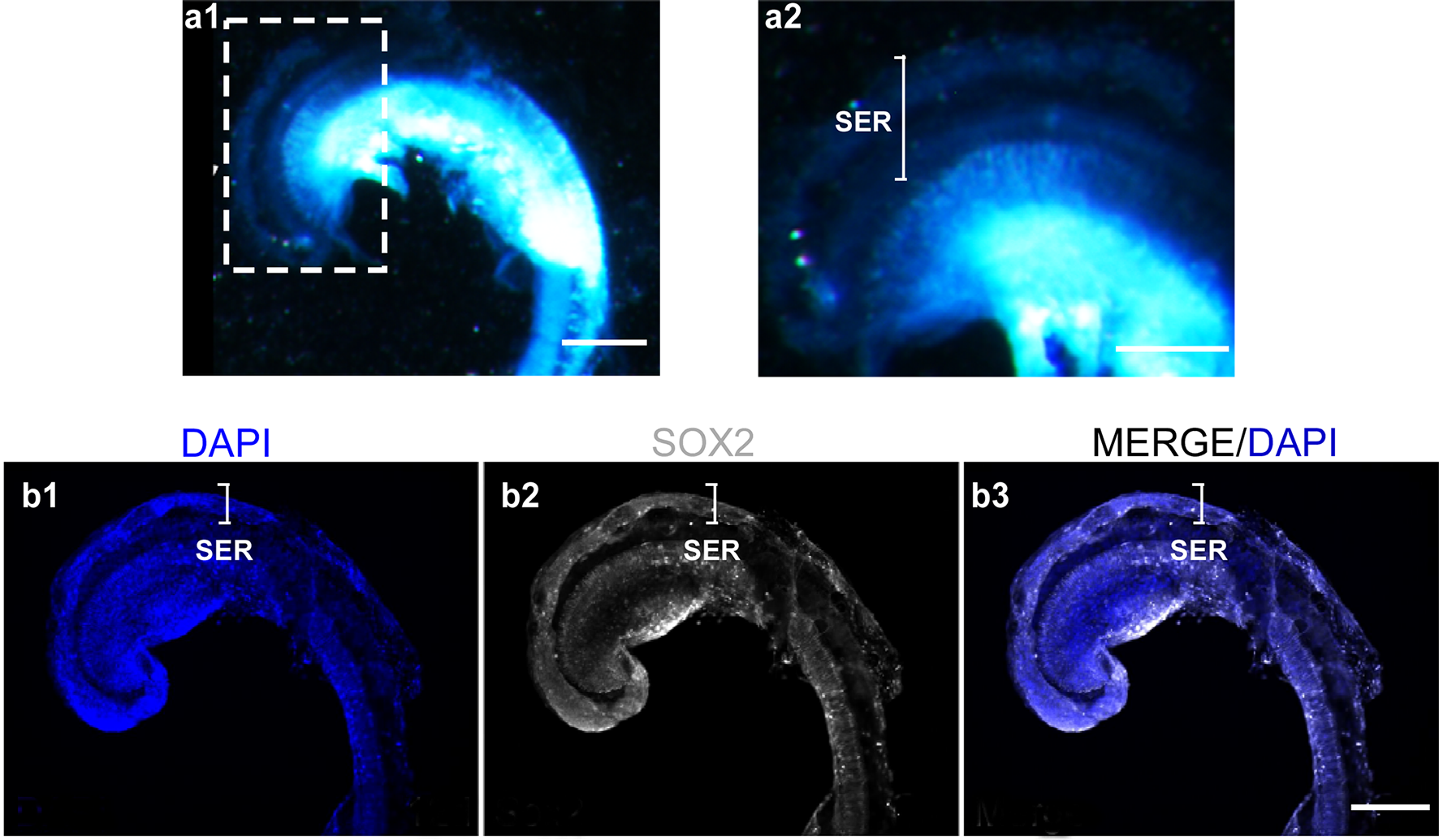

Supplement: Supplementary Figure 1 — Adult cochlear sensory epithelium degenerates in culture with the conventional method. (a1,a2) Epifluorescent picture of the apical turn of the freshly dissected wild-type adult mouse cochlea according to the protocol established for culturing neonatal mouse cochlea (Parker et al., 2010; Landegger et al., 2017). Only the very apical region of the sensory epithelium could be preserved with this method. Panel (a2) is the box area in panel (a1), with the sensory epithelial regions (SER) marked with a bracket. (b1–b3) Fluorescent picture of the apical turn of an adult mouse cochlea using the traditional culture method. After 2 weeks in culture, the whole sensory epithelium folded and degenerated, with the loss of virtually all SOX2+ cells. Scale bars: 50 μm. [file Image_1.TIF]

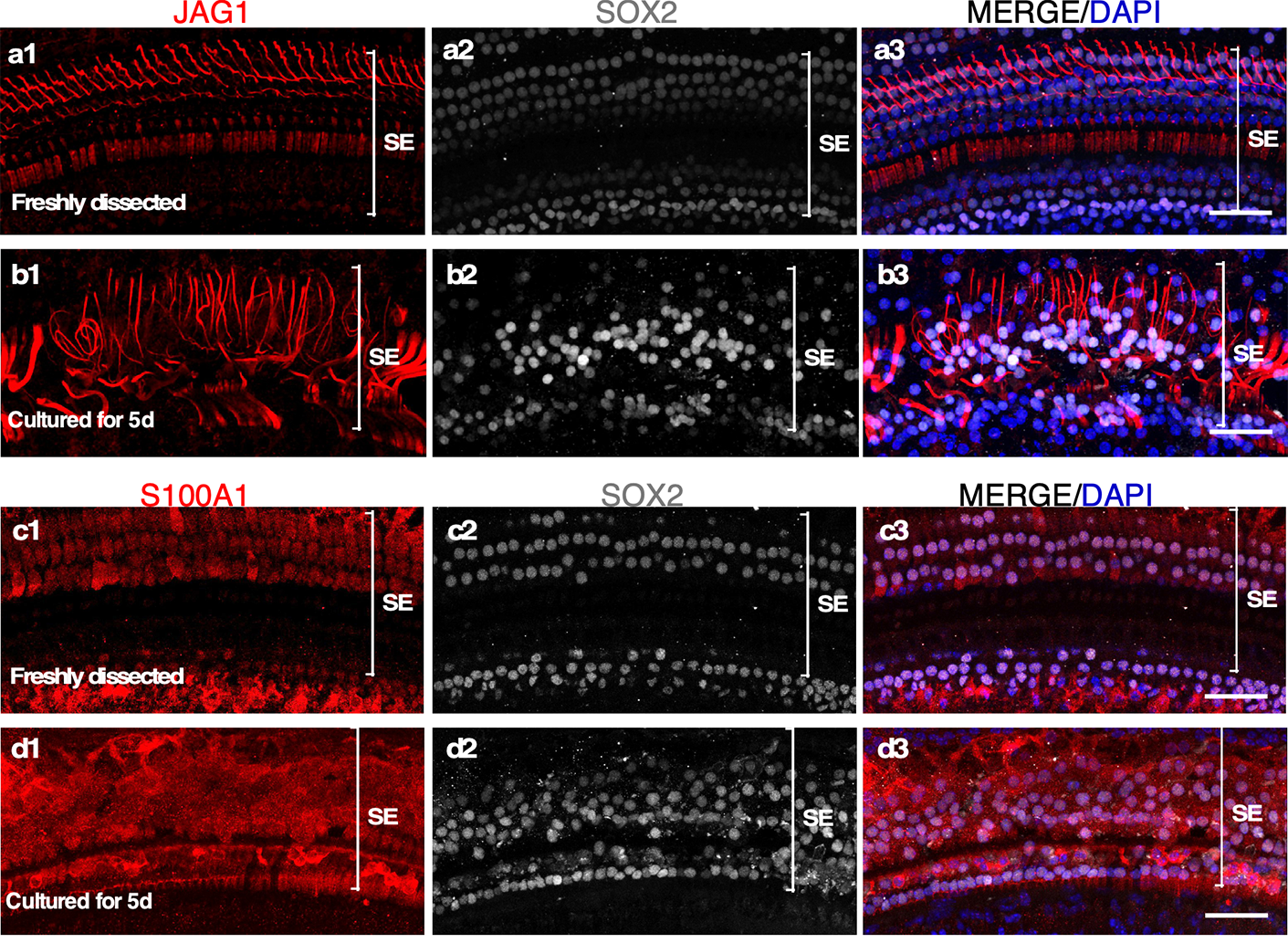

Supplement: Supplementary Figure 2 — Maintenance of supporting cell (SC) markers in cultured adult cochleae. (a1–a3) In a freshly dissected adult cochlea (middle turn), markers SOX2 and JAG1 were co-localized in the SCs. (b1–b3) After 5 days in culture, a subset of SOX2+ cells was co-labeled with JAG1. Notice disorganization of SCs compared with the freshly dissected sample. (c1–c3) Co-localization of SOX2 and S100A1 in a subset of SCs of a freshly dissected adult cochlea. (d1–d3) SOX2 and S100A1 were similarly co-localized in a subset of SCs of adult cochleae after 5 days in culture. Again, the disorganization of SCs was evident in culture. Scale bars: 50 μm. [file Image_2.TIF]

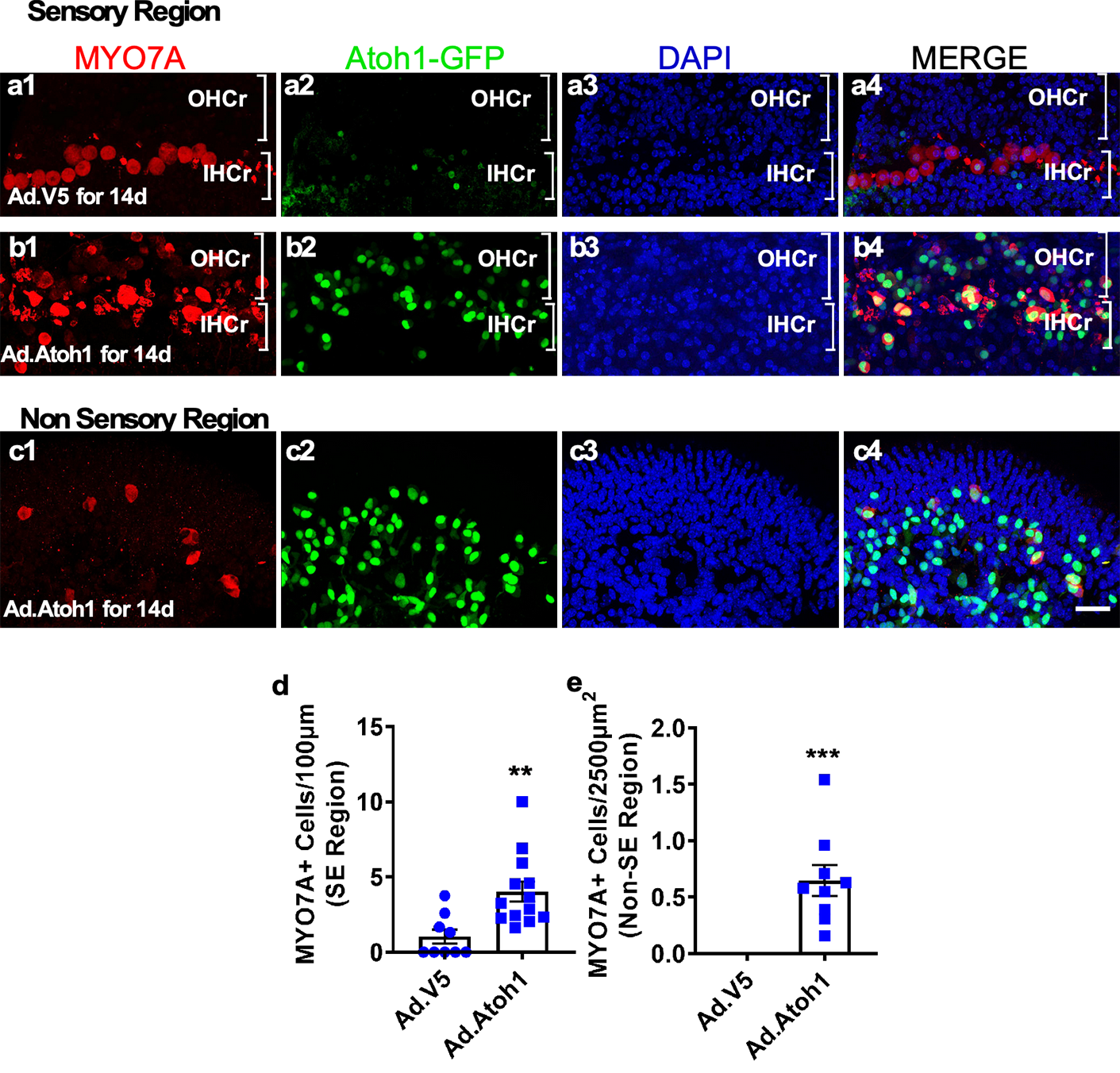

Supplement: Supplementary Figure 3 — In vitro regeneration of hair cell-like cells (HCLCs) with Atoh1 overexpression. (a1–a4) In cultured adult Atoh1-GFP cochleae infected with Ad-V5, a few GFP+ cells were observed mainly in the existing inner HCs (IHCs), but not in other sensory epithelial regions. In cultured adult Atoh1-GFP cochleae infected with Ad-Atoh1, increased numbers of GFP+ cells were observed across the sensory epithelial region (b1–b4) and the limbus region (c1–c4). Some GFP+ cells were co-labeled with MYO7A, indicating regeneration of HCLCs in those areas. (d,e) Quantification data showed more MYO7A+ HCLCs that were detected in both the sensory epithelial region and the limbus region in cultured adult Atoh1-GFP cochleae infected by Ad-Atoh1 than by Ad-V5 infection. **p < 0.01, ***p < 0.001, Student’s t-test. Error bar, mean ± SEM, n = 9–13. n is the number of biologically independent cochlea samples. Scale bars: 20 μm. [file Image_3.TIF]

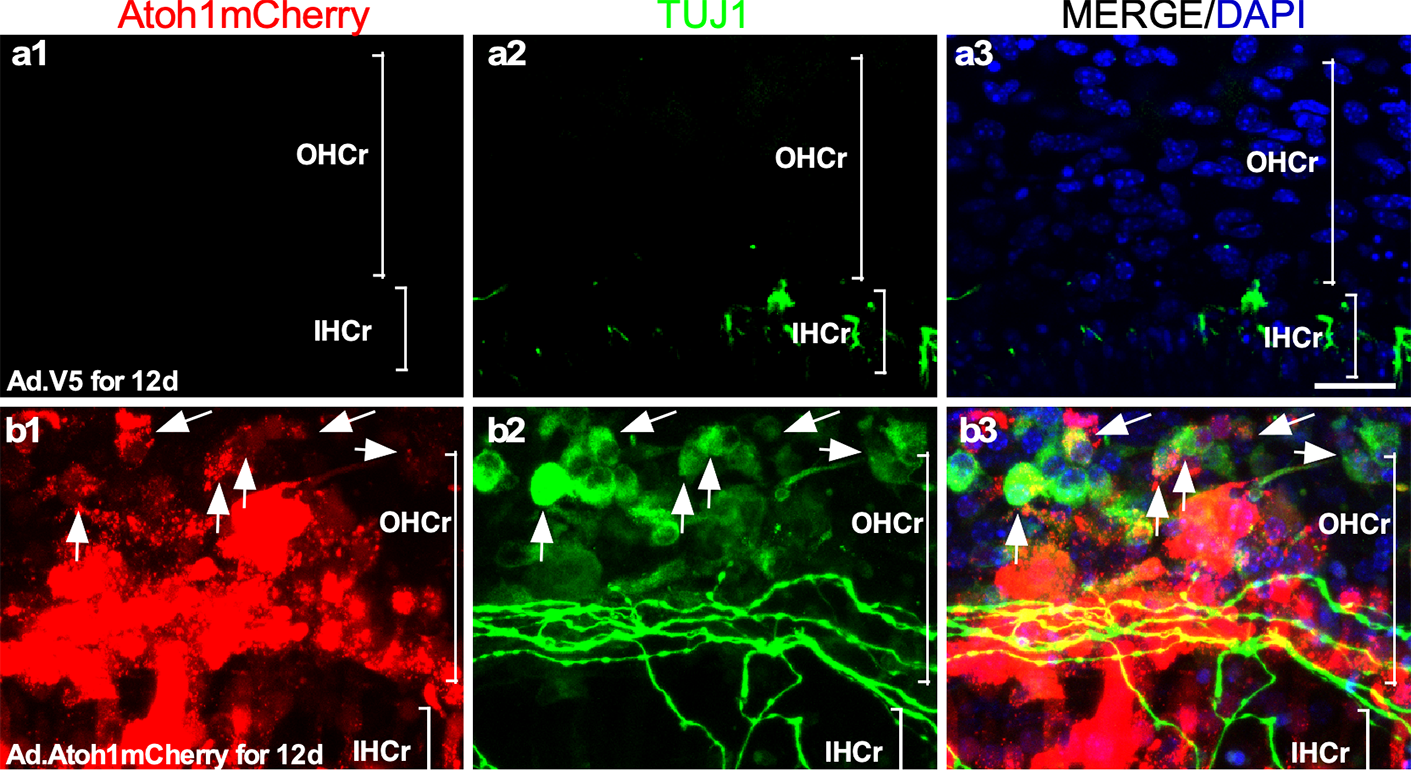

Supplement: Supplementary Figure 4 — Atoh1 overexpression generates TUJ1+ cells in adult cochlear explant culture. (a1–a3) Nine days postkanamycin/furosemide injection in vivo and 12 days after Ad-V5 infection in culture (d12), and there was a loss of a majority of neurites in the IHC region (IHCr, bracket). (b1–b3) Nine days postkanamycin/furosemide injection in vivo and 12 days after Ad-Atoh1-mCherry infection in culture, adult cochleae showed regenerated TUJ1+/mCherry+ cells in the OHC region (OHCr, bracket), and significantly more neurites in the IHC region. TUJ1+ cells were co-labeled with mCherry (arrows). Scale bars: 20 μm. [file Image_4.TIF]

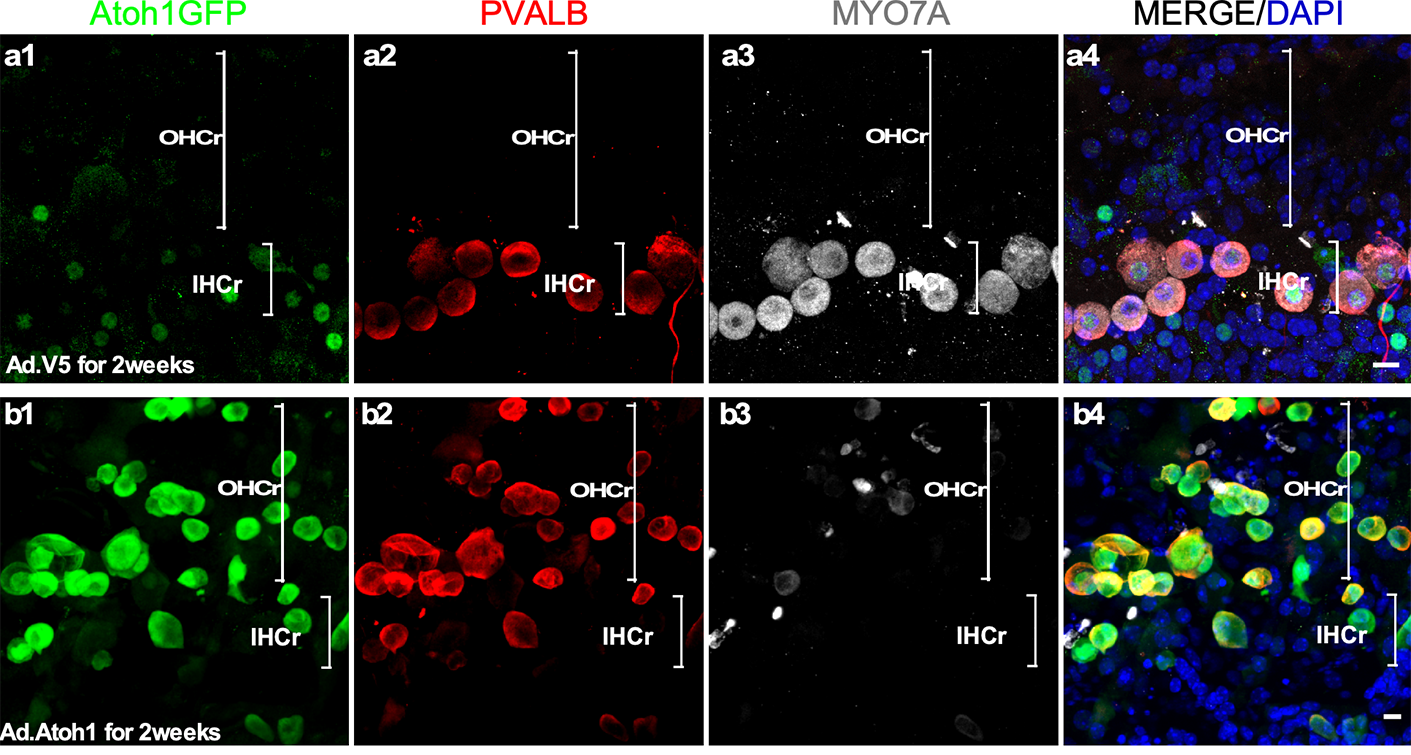

Supplement: Supplementary Figure 5 — In vitro regeneration of HCLCs with Atoh1 overexpression. Enlarged picture of the apex of a cultured adult Atoh1-GFP cochlea infected with Ad-V5/Ad-Atoh1. The samples were incubated with antibodies against GFP/MYO7A/PVALB. (a1–a4) In cultured adult Atoh1-GFP cochleae infected with Ad-V5 (the enlarged image of Supplementary Figure 3a), a few weak GFP+ cells were observed mainly inside and surrounding the existing IHCs, indicating the weak endogenous ATOH1 activity. (b1–b4) Many strong GFP+ cells were observed across the sensory epithelial region. Most GFP+ cells were co-labeled with PVALB. Scale bars: 10 μm. [file Image_5.TIF]

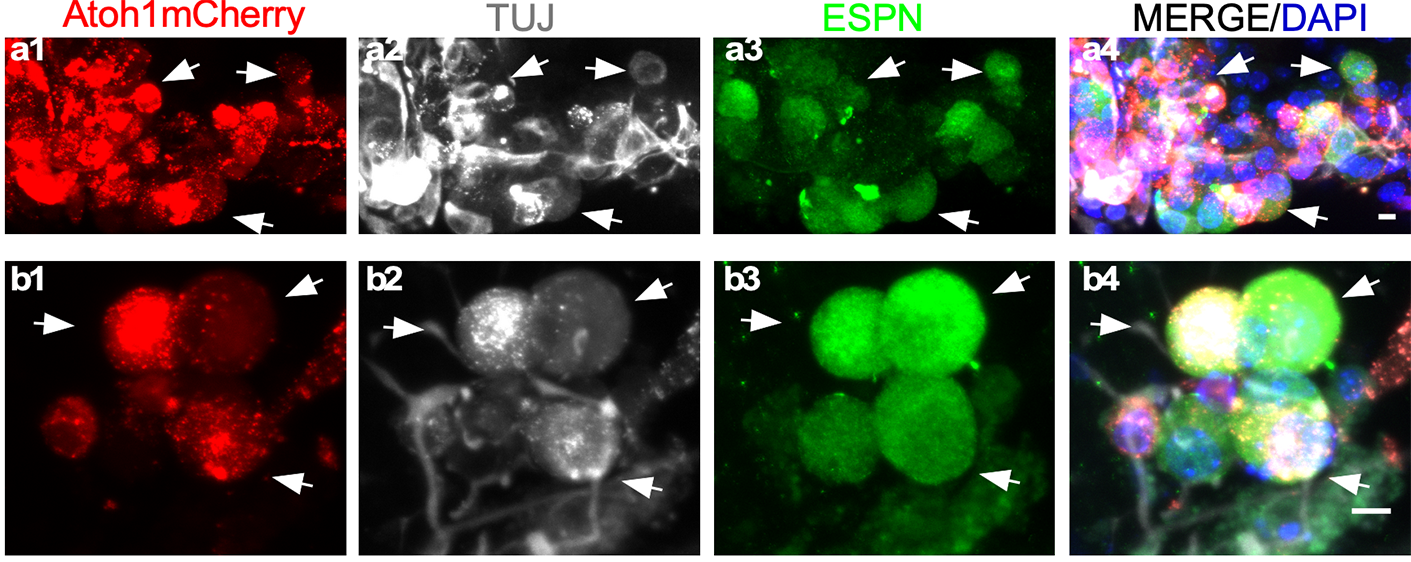

Supplement: Supplementary Figure 6 — Atoh1 overexpression generates TUJ1+/ESPN+ HCLCs in adult cochlear explant culture. (a1–a4) (Low magnification) and (b1–b4) (high magnification). Twelve days after Ad-Atoh1-mCherry infection of cultured WT adult cochleae, regenerated mCherry+/TUJ1+/ESPN+ HCLCs in the sensory region of apex were detected. Atoh1mCherry+/TUJ1+ cells were co-labeled with an HC marker ESPN (arrows). Scale bars: 10 μm. [file Image_6.TIF]
