# Supplementary material for: A Novel in vitro Model Delineating Hair Cell Regeneration and Neural Reinnervation in Adult Mouse Cochlea
Source: Front Mol Neurosci. 2022 Jan 10;14:757831. doi: 10.3389/fnmol.2021.757831 (PMC8785685; doi:10.3389/fnmol.2021.757831)
Supplement: Supplementary file 7 [file Data_Sheet_1.docx]

**Video S1.** The dissection process of the adult cochlea in HBSS medium with fine tip forceps.

**Video S2.** The Maintenance of architecture of cultured cochlea (Left: Freshly dissected; Right: Cultured for 14 days)

**Video S3 (IHC-neurites.mp4). 3D-reconstructed movie for the region in the dotted box in Fig 8 d3 showing the connections between IHCs and neurites.**

**Video S4 (OHC-neurites.mp4). 3D-reconstructed movie for the region in the densely dotted box in Fig 8 d3 showing the connections between OHC and neurites.**
