# Supplementary material for: A Novel in vitro Model Delineating Hair Cell Regeneration and Neural Reinnervation in Adult Mouse Cochlea
Source: Front Mol Neurosci. 2022 Jan 10;14:757831. doi: 10.3389/fnmol.2021.757831 (PMC8785685; doi:10.3389/fnmol.2021.757831)
Supplement: Supplementary file 8 [file Table_1.DOCX]

| **Antibodies** | **Species** | **Dilution** | **Source** | **Cat#** |
| --- | --- | --- | --- | --- |
| Acetylated tubulin (Ac-TUBA4A) | Mouse | 1:500 | Sigma-Aldrich | T6793 |
| ESPN | Rabbit | 1:200 | Gift from J. Bartles | Northwestern Univ |
| JAG1 | Rabbit | 1:200 | Santa Cruz Biotech | sc8303 |
| MYO7A | Rabbit | 1:500 | Proteus Biosciences | 25-6790 |
| PVALB | Mouse | 1:500 | Sigma-Aldrich | P3088 |
| PTPRQ | Rabbit | 1:200 | Gift from Dr. Bowen-Pope | Univ of Washington |
| S100A1 | Rabbit | 1:200 | Sigma-Aldrich | HPA006462 |
| SOX2 | Goat | 1:200 | Santa Cruz Biotech | sc-17320 |
| TUJ1 **(**Anti-beta III Tubulin**)** | Mouse | 1:200 | Abcam | Ab78078 |

All Alexa Fluor secondary antibodies were purchased from Invitrogen, with 1:1000 dilution. Alexa Fluor^TM^ 488 Phalloidin (F-Actin) was purchased from Thermo Fisher Scientific (A12379), with 1:500 dilution.
